# Refugees, war trauma and mental health: knowledge and experience from trainees and early career psychiatrists

**DOI:** 10.1192/j.eurpsy.2023.639

**Published:** 2023-07-19

**Authors:** S. Bianchi, I. Riboldi, D. Őri, S. Pompili, C. Pavel, A. Seker, E. Psychiatry Across Borders Working Group

**Affiliations:** ^1^Department of Psychiatry, University of Perugia, Perugia; ^2^Department of Medicine and Surgery, University of Milano-Bicocca, Monza, Italy; ^3^ Institute of Behavioural Sciences, Semmelweis University; ^4^Department of Mental Health, Heim Pal National Pediatric Institute, Budapest, Hungary; ^5^Clinical Unit of Psychiatry, Department of Neuroscience, University Polytechnic of Marche, Ancona, Italy; ^6^Prof. Dr. Alexandru Obregia Clinical Psychiatry Hospital, Bucharest, Romania; ^7^Department of Child and Adolescent Psychiatry, Institute of Psychiatry, Psychology and Neuroscience, King’s College London, London, United Kingdom; ^8^ European Federation of Psychiatric Trainees

## Abstract

**Introduction:**

Psychiatry Across Borders working group of the European Federation of Psychiatric Trainees has the mission to improve psychiatric training in transcultural psychiatry and trauma-related topics in Europe. It started conducting a survey in 2016, to assess trainees’ experiences in forcibly displaced people mental health **(**Frankova *et al.* Transcult.Psychiatry, in press).

**Objectives:**

To investigate European psychiatric trainees’ and Early Career Psychiatrists’ (ECPs) training about trauma and refugees’ mental health, focusing on educational and clinical difficulties occurring while assisting war refugees or in Eastern Europe, due to the ongoing conflict.

**Methods:**

A new survey for European psychiatric trainees and ECPs was designed. A web questionnaire was shared through various channels, including social media, in September 2022. It included an informed consent form and investigated socio-demographic data, training in trauma and refugees’ mental health, clinical practice in war areas or with war refugees.

**Results:**

As of 16/10/22, 31 were the responders, mainly adult psychiatrists (93.6%). Although the 87.1% worked with forcibly displaced people, only 29% received a specific training, and 53.6% didn’t feel prepared to face war trauma-related disorders. However, 64.3% could reach out to a teamwork member specialized in the topic, and 72.2% to interpreters. The 67.7% worked with actual war refugees, mainly addressed to psychiatric services due to new onset of psychiatric symptoms, especially insomnia (66.7%), often diagnosed with Acute Stress Reaction (66.7%) and treated with psychiatric drugs (83.3%). Two colleagues working in war areas participated in the survey: both lost patients at follow-up and experienced increased workload or lack of means (i.e., drug supply) or support.

**Conclusions:**

This survey can spot educational needs in transcultural psychiatry, helping to program targeted interventions for psychiatric trainees and ECPs, aimed at implementing clinical practice towards the common goal of social cohesion.

**Disclosure of Interest:**

None Declared

